# The contribution of *Saccharomyces cerevisiae* replicative age to the variations in the levels of Trx2p, Pdr5p, Can1p and Idh isoforms

**DOI:** 10.1038/s41598-017-13576-w

**Published:** 2017-10-16

**Authors:** Aglaia V. Azbarova, Kseniia V. Galkina, Maxim I. Sorokin, Fedor F. Severin, Dmitry A. Knorre

**Affiliations:** 1 Faculty of Bioengineering and Bioinformatics, Moscow State University, Leninskiye Gory 1-73, Moscow, 119991 Russia; 2 Belozersky Institute of Physico-Chemical Biology, Moscow State University, Leninskiye Gory 1-40, Moscow, 119991 Russia; 3 National Research Centre Kurchatov Institute, Centre for Convergence of Nano-, Bio-Information and Cognitive Sciences and Technologies, Moscow, 123182 Russia; 4 OmicsWay Corp., 340S Lemon Ave, Walnut, CA, 91789 USA

## Abstract

Asymmetrical division can be a reason for microbial populations heterogeneity. In particular, budding yeast daughter cells are more vulnerable to stresses than the mothers. It was suggested that yeast mother cells could also differ from each other depending on their replicative age. To test this, we measured the levels of Idh1-GFP, Idh2-GFP, Trx2-GFP, Pdr5-GFP and Can1-GFP proteins in cells of the few first, most represented, age cohorts. Pdr5p and Can1p were selected because of the pronounced mother-bud asymmetry for these proteins distributions, Trx2p as indicator of oxidative stress. Isocitrate dehydrogenase subunits Idh1p and Idh2p were assessed because their levels are regulated by mitochondria. We found a small negative correlation between yeast replicative age and Idh1-GFP or Idh2-GFP but not Trx2-GFP levels. Mitochondrial network fragmentation was also confirmed as an early event of replicative aging. No significant difference in the membrane proteins levels Pdr5p and Can1p was found. Moreover, the elder mother cells showed lower coefficient of variation for Pdr5p levels compared to the younger ones and the daughters. Our data suggest that the levels of stress-response proteins Pdr5p and Trx2p in the mother cells are stable during the first few cell cycles regardless of their mother-bud asymmetry.

## Introduction

Some microorganisms show significant levels of individual cells heterogeneity^1–3^. This could be due to DNA replication errors and subsequent proliferation of the new subclones within the population^4^. However, there can also be non-genetic sources of the heterogeneity. Some factors that are present in the cells at very low copy numbers^5^ can be unevenly distributed during the cell divisions. For instance, an inequality in the inherited mitochondria can render differences between the individual cells^6^. A switch of aggregation-prone proteins to prion states was also shown to be a source of phenotypic heterogeneity in yeast populations^7^. Another source of cell-to-cell variability are cycling processes. The low-frequency localization pulsing of stress-response transcription factors was reported in yeast^8^. Finally, the concentrations of many mRNAs and proteins are also fluctuating due to subsequent activation of the factors specific to the cell cycle stages^9,10^.

Additionally, asymmetrical cell division is an important mechanism of mother and daughter cells diversification. For instance, it was shown that in stationary phase *Saccharomyces cerevisiae* mother and daughter cells segregate into the populations of ‘nonquiescent’ and ‘quiescent’ cells^3,11^. Moreover, baker’s yeast cells have the limited capacity for budding^12^. This phenomenon is usually referred to as “replicative aging”. It was shown that after 15–20 divisions mother cells reach a senescence entry point and then die^13,14^. However, due to the dilution by the newborn cells the proportion of such replicatively old ones is extremely low (see for instance^15^). At the same time, some changes were reported for relatively young yeast ‘cohorts’, which are the mostly represented ones in yeast populations. First, Kale & Jazwinski^16^ have shown that the resistance to genotoxic stresses is different between yeast mother cells of relatively young ‘cohorts’. Next, the change of mitochondrial morphology was reported for yeast cells of replicative age 5–9^17^. We have also shown recently that the resistance to acetic acid stresses and heat shock is higher in yeast mother cells within replicative age 2–4^18^ compared to that of the daughter cells or the older mothers. We found that the percentage of cells with distinguishably different mitochondrial populations is enriched in yeast cells with age  ≥5^19^. Finally, it was shown that aggregates containing Hsp104 accumulate in yeast cells which have already produced 3–6 buds, while being absent in the newborn cells^20^. According to these observations, it was suggested that the replicative age provides additional source for cell variability^21^.

If this is indeed the case, yeast mother cells of highly represented age ‘cohorts’ (i.e. with 1–9 bud scars) are expected to carry significantly different concentration of several proteins. We reasoned that, as the level of stress-induced damage increases with the replicative age, the abundances of stress-response proteins are also likely to correlate with the age. To test this hypothesis we selected three stress-response proteins: (1) mitochondrial isocitrate dehydrogenases Idh1p, Idh2p which levels are regulated by mitochondria-to-nucleus signaling pathway, activated by dysfunctional mitochondria^22–24^ and (2) cytoplasmic thioredoxin Trx2p. *TRX2* gene is regulated by H_2_O_2_-sensor transcription factor Yap1p^25^, thus Trx2p concentration in individual cell reflects the level of oxidative stress response activation.

We have also selected two other proteins, Pdr5p and Can1p, mostly because they are supposed to show a significant asymmetry in mother-daughter distribution. Pdr5p is a plasma membrane pleiotropic drug resistance ABC-transporter, its expression is regulated by transcription factors *PDR1* and *PDR3* ^26,27^. These transcription factors are activated, among others, in response to mitochondrial dysfunction^28^. Changes of mitochondria functioning are one of the earliest manifestations of yeast aging^17,19^, so we reasoned that this protein is likely to be accumulated in mid-aged mother cells. Can1p is arginine permease, it has been recently found to be one of the key regulators of yeast replicative lifespan^29^.

Thus, we measured the levels of Trx2-GFP, Idh1-GFP, Idh2-GFP, Pdr5-GFP and Can1-GFP in the yeast cells of different replicative age cohorts. As a positive control for our method, we have also tested the heterologously expressed fragment of human huntingtin 103Q-CFP. This protein is known to form aggregates, which in yeast tend to remain in the mother cells^30^. We found that while Trx2-GFP, Pdr5-GFP, Can1-GFP did not show any significant variation with mother cells age, the levels of Idh1-GFP\Idh2-GFP and 103Q-CFP showed negative and positive correlations correspondingly.

## Results

**Figure 1:**
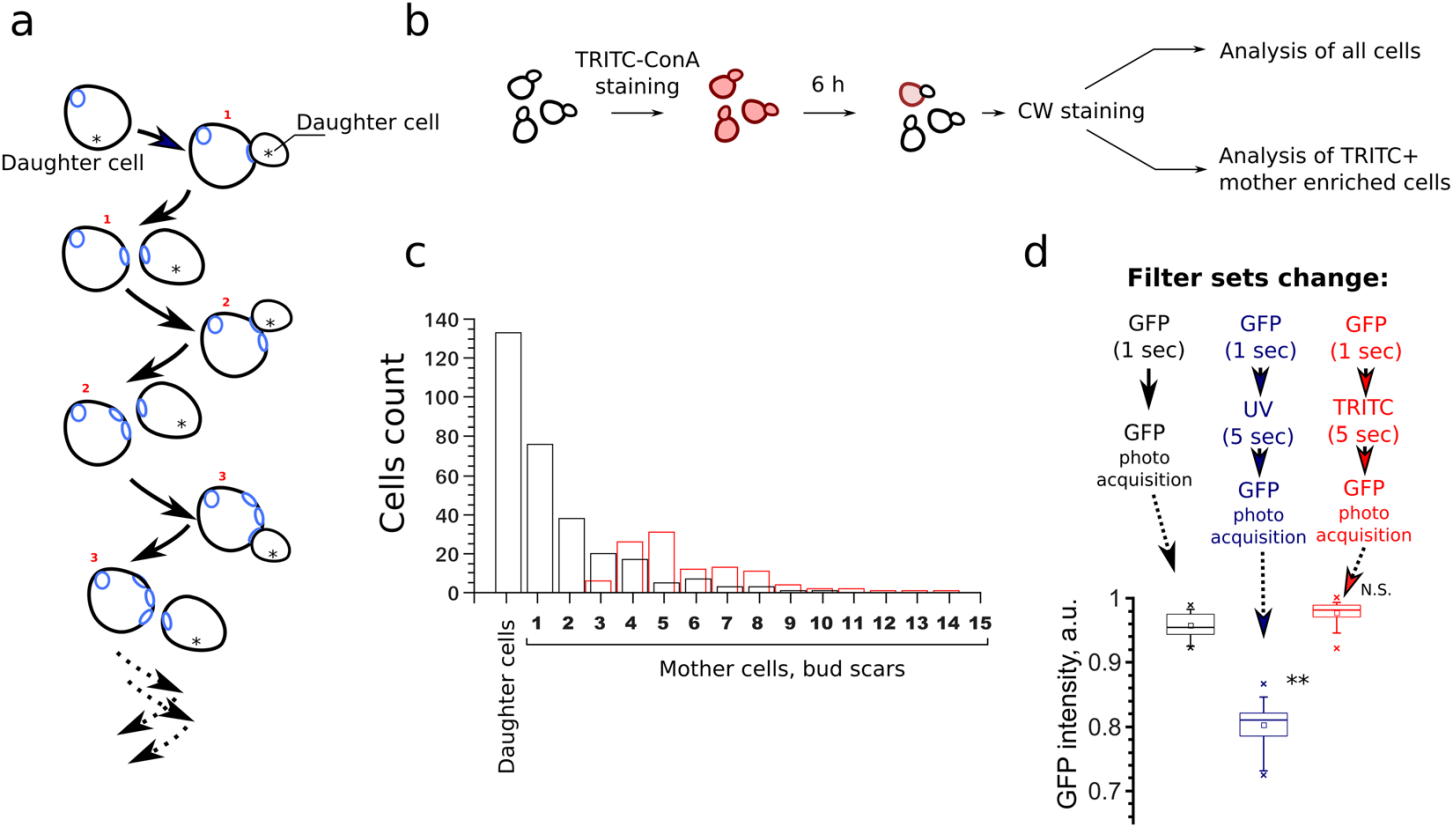
Experimental approach. (**a**) Yeast cell division cycles result in the formation of the mother cells with different numbers of chitin enriched bud scars. Asterisks indicate the buds (daughter cells), digits - the replicative age (number of bud scars) of mother cells. (**b**) An outline of the performed experimental procedures. Exponentially growing yeast cells were stained with TRITC-ConA, then grown for 6 hours, stained with CW (Calcofluor white) and analyzed. In each biological replicate the first 40–60 randomly selected cells were photographed in GFP- and then CW-channel (analysis of all cells), afterwards 40–60 TRITC-positive cells from the same sample were selected and photographed in the same channels. (**c**) Distribution of yeast cells in the replicative aging cohorts (black bars). The distribution of TRITC-ConA enriched mother cells (red bars). (**d**) The ratio of Trx2-GFP fluorescence intensity before and after the illumination with U-MNU2 (UV, excitation wavelength λ = 360–370 nm) and U-MNG2 (TRITC, excitation wavelength λ = 530–550 nm) filter sets. **P value < 0.01 according to Wilcoxon rank-sum unpaired test.

Asymmetrical division of yeast cells allow to attribute a replicative age (the number of produced buds) to each cell (Fig. 1a). The number of cells in each replicative age cohort is approximately a half of the number of previous (younger) replicative age cohort. In addition to the biological significance of this fact, it makes it technically difficult to find in yeast suspension the cells of replicative age higher than five. To increase sample size of mothers cells with age 5–9 we stained yeast cells with TRITC-ConA and allowed them to perform 4–5 divisions afterwards (Fig. 1b, see materials and methods). TRITC-ConA positive cells were substantially enriched by mother cells with 4–8 bud scars (Fig. 1c). Therefore, in each experiment in addition to whole population of yeast cells we analyzed a fraction of TRITC-ConA positive cells. The age of yeast cells was analyzed by the number of bud scars visualized by Calcofluor White (CW), while the levels of proteins were estimated by the average intensity of GFP in cells (see methods section for details). Importantly, we calculated bud scars of the cells only after they were photographed in GFP-channel. Indeed, even short illumination of yeast cells with UV-light (U-MNU2 filter set, required for CW staining) bleached GFP, while that was not the case for green-light (U-MNIBA3 filter set, required for TRITC-ConA staining, see Fig. 1d).

**Figure 2:**
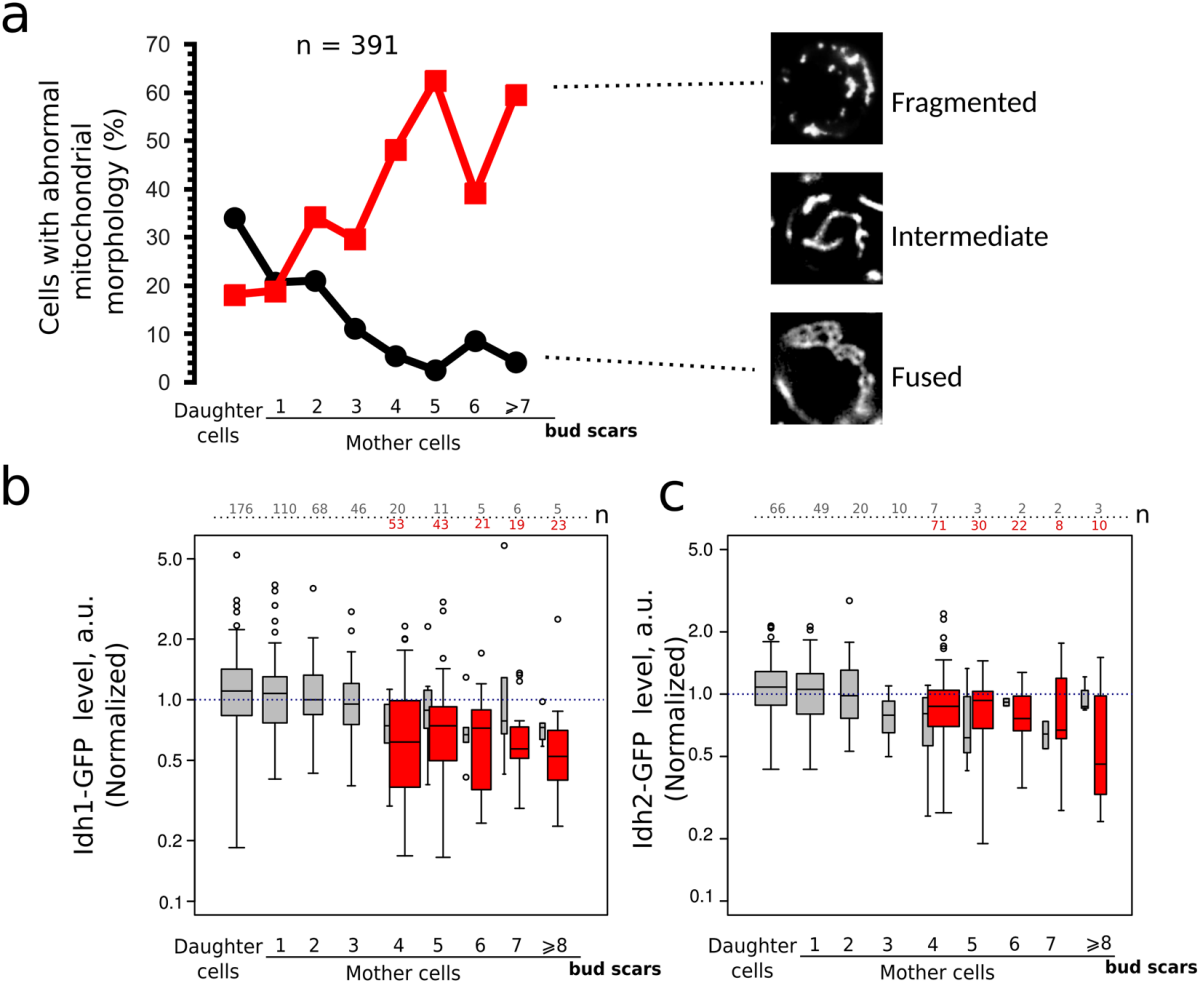
The levels of Idh1-GFP and mitochondrial morphology in yeast cells correlate with the age of the mother cells. (**a**) The percentage of yeast cells with fragmented or fused mitochondrial network morphology. The data (391 cells) were pooled from four separate day experiments. Correlation coefficient (Kendall’s tau) for yeast age and percentage of yeast cells with fragmented mitochondria is equal to 0.714 (P = 0.014). The difference between the age cohorts of the mother cells is significant, p-value = 0.00018 according to Pearson’s Chi-squared test with simulated p-value. The representative photographs of different types mitochondrial morphology networks visualized by mitochondrial protein Idh1-GFP are shown in the right. Idh1-GFP (**b**) and Idh2-GFP (**c**) levels in individual cells of different replicative age cohorts. Grey boxplots represent the random samples of yeast cells. There is a significant negative correlation between yeast mother cells age and Idh1-GFP level (see Table 1). Red box plot represents TRITC+ (age > 4) enriched mother cells. The numbers of analyzed cells for each age class are shown at the top of the boxplot. The bin size for TRITC+ and TRITC− cells are designated separately.

Is it possible to explain heterogeneity of yeast clonal cultures by the replicative age dependent accumulation of some proteins in mother cells? To test this we measured the levels of the proteins chosen for the aforementioned reasons in the yeast age cohorts. We suggested earlier that accumulation of dysfunctional mitochondria can be the reason for the early decline of the stress resistance in yeast cells^19^. Therefore, as the first step we compared the mitochondrial morphology and the levels of Idh1-GFP protein in the different replicative age cohorts. *IDH1* is regulated by retrograde mitochondria-to-nucleus signaling pathway^31^. We noticed that in accordance with the results of Lam *et al*.^17^, there is a correlation between the yeast age and mitochondrial reticulum morphology (Fig. 2a). While the age positively correlated with the percentage of the cells with fragmented mitochondria the proportion of yeast cells with the hyperfused morphology decreased with age (Fig. 2a). At the same time we found a significant negative correlation between yeast mother cell replicative age and the level of Idh1-GFP (Table 1, Fig. 2b). Same correlation of age and protein level was found for another isoform of isocitrate dehydrogenase Idh2 (Table 1, Fig. 2c).

**Table 1:** Mother-bud asymmetry and the correlation of protein levels with replicative age of the mother cells.

Protein and conditions	Correlation of protein level with replicative age of mothers. Kendall’s tau (NS — *non significant*)	Mother/bud asymmetry (ratio of the means, p-value according to Wilcoxon Mann Whitney two-tailed test)
Idh1-GFP	−0.149^*^ (p = 0.000976)	**1.01** p = 0.206
Idh2-GFP	−0.197^*^ (p = 0.01082)	**0.88 p =**0.009253
Trx2-GFP	0.078^NS^	**1.18** p = 3.5 × 10^−6^
Trx2-GFP, H_2_O_2_	0.01^NS^	**1.28** p = 0.048
Pdr5-GFP	0.07^NS^	**1.38** p = 2 × 10^−5^
Pdr5-GFP, clotrimazole	0.076^NS^	**0.97** p = 0.66
Can1-GFP	0.049^NS^	**1.49** p = 10^−16^
103Q-CFP	0.11^*^ (p = 0.02656)	**1.76** p = 10^−16^

**Figure 3:**
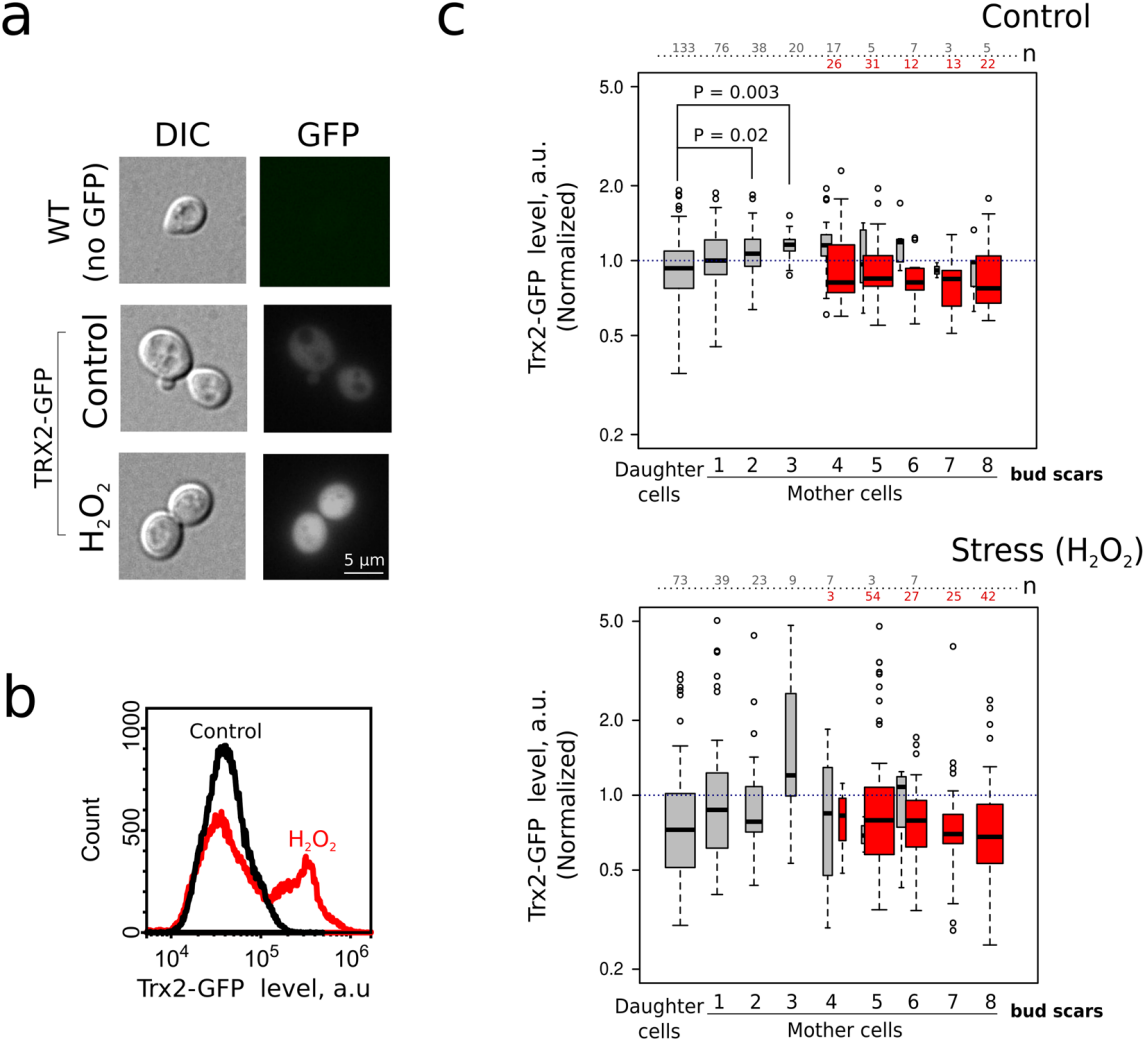
Trx2-GFP level displays mother-daughter asymmetry but does not differ between the mother cells of different ages. (**a**) Representative photograph of control yeast cells and yeast cells expressing Trx2-GFP. Treatment of yeast cells with H_2_O_2_ (2 mM) induces an increase of Trx2-GFP levels; (**b**) Analysis of Trx2-GFP levels by flow cytometry under the control conditions and in the presence of 2 mM H_2_O_2_; (**c**) Quantification of Trx2-GFP in yeast cells of different age cohorts under the control conditions (upper panel) or treated with H_2_O_2_. Red box plots indicate Trx2-GFP levels in TRITC-ConA positive (age > 4 enriched) mother cells. The numbers of analyzed cells for each age class are shown at the top of the boxplot. The bin size for TRITC+ and TRITC− cells are designated separately. The only detected difference was between the untreated daughter and the mother cells (*P < 0.05; **P < 0.005 according to Nemenyi test).

The mitochondrial dysfunction could be a reason for redox imbalance and oxidative stress^32^. Increased hydrogen peroxide levels in yeast cytoplasm induce relocalization of transcription factor Yap1p and subsequent activation of the target genes, in particular, *TRX2* ^33,34^. To assess Yap1p activation we measured Trx2-GFP levels in yeast cells (Fig. 3a). We expected that replicative age-dependent mitochondrial dysfunction can be manifested in differential activation of Yap1p depending on the replicative age of a cohort. Indeed, we found that only a portion of cells increased Trx2-GFP levels in response to exogenous hydrogen peroxide (Fig. 3b). However, the analysis of the different age cohorts showed that there was no statistically significant difference in Trx2-GFP levels, with the exception of mother-to-bud asymmetry in the absence of hydrogen peroxide (Fig. 3c, Table 1).

**Figure 4:**
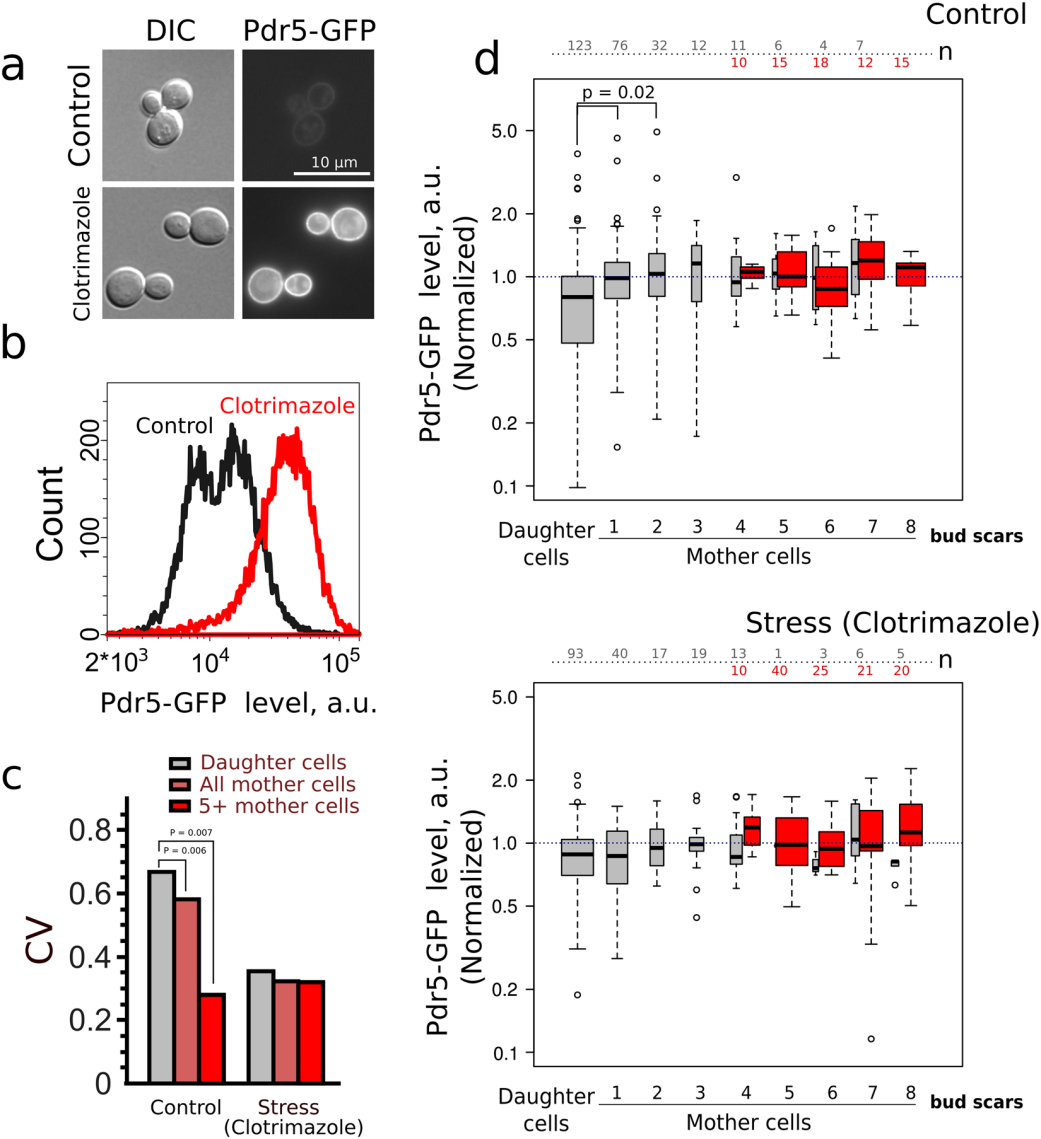
The level of Pdr5-GFP in yeast cells does not differ in the mother cells aging cohorts. (**a**) A representative photograph of the control and clotrimazole treated (20 μM) cells. (**b**) Quantification of Pdr5-GFP levels increase induced by clotrimazole. (**c**) Cell-to-cell coefficient of variation (CV) of Pdr5-GFP levels for daughter cells, all mother cells and TRITC+ (age > 4 enriched) mother cells with replicative age above five. P-value was calculated with Fligner-Killeen (median) test. (**d**) Pdr5-GFP levels in individual cells of different yeast cells replicative age cohorts. Grey boxplots represent the random samples of yeast cells, the red box plot represents TRITC+ cells. The control and the stress conditions (20 μM clotrimazole) were analyzed. The numbers of analyzed cells for each age class are shown at the top of the boxplot. The bin size for TRITC+ and TRITC− cells are designated separately. P value was calculated by Nemenyi-Test.

**Figure 5:**
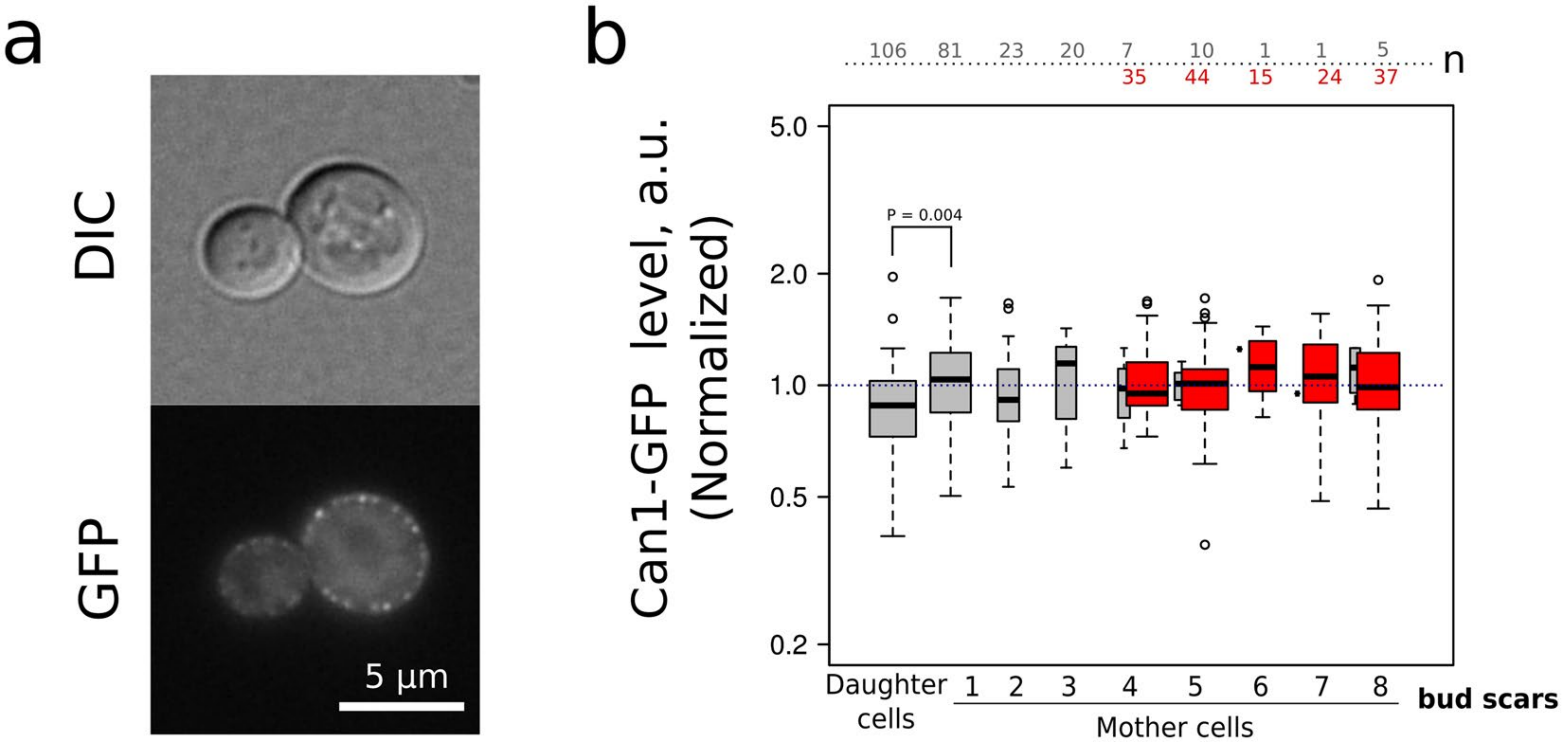
The level of Can1-GFP does not differ in the mother cells aging cohorts. (**a**) Representative photograph of the control yeast cells expressing Can1-GFP. (**b**) Can1-GFP levels in the individual cells of different replicative age cohorts (n = 410 cells). Grey boxplots represent the random samples of yeast cells, the red box plot represents TRITC+ (age > 4 enriched) mother cells. The numbers of analyzed cells for each age class are shown at the top of the boxplot. The bin size for TRITC+ and TRITC− cells are designated separately. P value was calculated according Nemenyi-Test.

Next, we tested the levels of major pleiotropic drug resistance pump Pdr5p. Functional Pdr5p is localized in plasma membrane, therefore we analyzed only the border area of the cell (see Figure S1). Clotrimazole is an inducer of *PDR5* gene^35^. Accordingly, we have shown that clotrimazole supplementation induces accumulation of Pdr5-GFP in yeast cells (Fig. 4a,b). To test the ability of yeast cells belonging to different age cohorts to activate mechanisms of antibiotic resistance we compared Pdr5-GFP levels and variance in the absence and in the presence of clotrimazole (Fig. 4c,d). Surprisingly, despite pronounced mother-to-bud asymmetry of Pdr5-GFP levels the Kruskal–Wallis H test did not show significant difference among cohorts of mother cells (see Table 1). Moreover, there was no significant correlation between mother cells age and Pdr5-GFP levels (see Table 1). Interestingly, we found that there is a significantly lower coefficient of variation of Pdr5-GFP levels in ‘mature’ (TRITC+) yeast mother cells than in overall mother cell population which is enriched with ‘young’ mother cells (Fig. 4c). Clotrimazole treatment did not induce age-specific accumulation of Pdr5-GFP (Fig. 4d, lower panel; Table 1). Similar results were obtained when we compared the levels of arginine permease Can1-GFP, the plasma membrane protein, in yeast of different age cohort. *CAN1* was recently identified in screening for most noisy *S.cerevisiae* genes^36^. However, despite the significant mother-to-bud asymmetry (Fig. 5, Table 1) we did not find any age-dependent variation of Can1-GFP levels.

**Figure 6:**
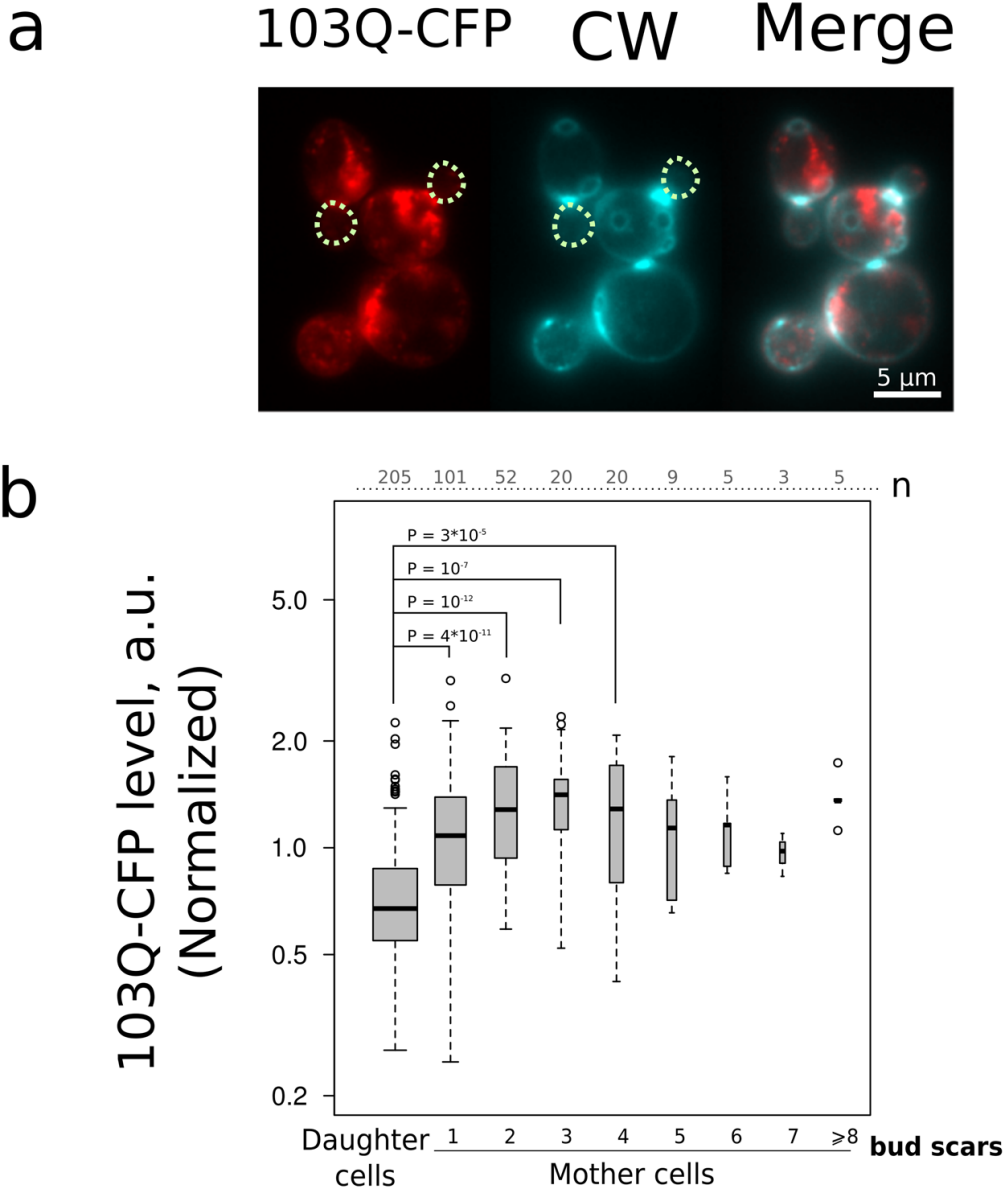
103Q-CFP level displays mother-daughter asymmetry. (**a**) Representative photograph of the group of cells expressing 103Q-CFP (red) stained with calcofluor white (blue). Image was generated by pseudo colouring: CFP-channel black and white image was copied to R channel, CW-channel image — to G and B channels of the RGB file. The dotted circles mark daughter cells. (**b**) Quantification of the average 103Q-CFP levels for replicative age cohorts. The numbers of analyzed cells for each age class are shown at the top of the boxplot. P value was calculated by Nemenyi-Test.

Apart from plasma membrane proteins, protein aggregates can be asymmetrically distributed between the mother cell and the bud. This asymmetry is a result of either constrained diffusion across the bud neck^37^ or an active directed transport of such aggregates^38^. We expected that the continuous protein synthesis can supply aggregates with additional components eventually making the old mother cells enriched by such aggregates if compared to ‘young’ mother cells. To test this possibility we used polyglutamine (103Q) fragment of human mutant huntingtin gene fused with cyan fluorescent protein (CFP)^39,40^. The gene was set under the regulation of P_GAL_ promoter. 103Q-CFP was expressed for 5 hours and assessed using fluorescent microscopy. The daughter cells were found to be mainly devoid of CFP aggregates (Fig. 6a, Table 1). As expected, we found a small positive correlation (Kendall’s Tau 0.11, P = 0.02) between replicative age and average intensity of CFP in cells (Table 1). Notably, this correlation arose mostly from the differences within the several first age cohorts of the mother cells (Fig. 1b). As 103Q-CFP was expressed for 5 hours (i.e. 3–4 cell divisions), there was no increase in CFP levels within mother cells of replicative age above four.

## Discussion

The age-dependent differentiation of asymmetrically divided microorganisms might contribute to optimization of their performance under stressful conditions. Indeed, it is suggested that heterogeneity of some traits can benefit microorganisms upon stress^36,41^. In the case of pathogens, the inhibition of such mechanisms could be a promising strategy to prevent the survival of the relatively resistant subpopulation. It was shown that old mother cells of some pathogenic yeast species are more resistant to antimycotics or host immune system. This leads to an increased proportion of the old cells in the host^42–44^. Understanding the mechanisms of yeast replicative aging is important not only because it is a model of mammalian aging but also because it can help to reveal the bet hedging strategies acting to increase the survival of the clonal population.

Baker’s yeast *S.cerevisiae* is a widely used model system for aging studies. It was found that some genetic modifications can change average replicative lifespan in the range of 20–30 generations^45–47^. However, yeast mother cells of replicative age above 20 are diluted by more than million of their descendants and thus are excluded from the selection pressure. Accordingly, few first replicative age cohorts represent the majority of the cells (see Fig. 1c). We suggested that the active mechanisms of cell components partitioning could cause significant differences between these ‘major’ mother cell cohorts. If so, the genesis of such differences could be an important mechanism of phenotypic heterogeneity in yeast clonal populations. In our work we compared the levels of several selected proteins to test this possibility. We found that the levels of mitochondrial protein Idh1 negatively correlated with replicative age of yeast exponentially growing population (Fig. 2). The increasing replicative age was also associated with the changes of mitochondrial morphology. The latter observation is in line with the work of Lam *et al*.^17^ who showed that the changes of mitochondrial morphology are one of the first manifestations of replicative aging. In the same work the authors found another early hallmark of aging — increased reactive oxygen species (ROS) levels (as detected by dihydroethidium bromide staining). An increase in the reactive oxygen species (ROS) production rate and the reshaping of mitochondrial network can induce each other. Indeed, on the one hand, dysfunctional mitochondria promote ROS accumulation in the cells^32^. On the other hand, ROS can induce depolarization of mitochondria^48,49^ in this way inhibiting mitochondrial fusion and promoting fragmentation of mitochondrial network^50,51^. We did not find any indications of early age-associated ROS increase — there was no correlation between Trx2-GFP levels and replicative age in our experiments (Fig. 3). Importantly, the Trx2-GFP levels were responsive to exogenously added hydrogen peroxide (Fig. 3a,b). Therefore, during the course of yeast replicative aging the reshaping of mitochondrial network takes place earlier than ROS accumulation.

The inhibition of mitochondrial fission increases the lifespan of yeast and filamentous fungi^52^. The latter is not surprising because mitochondrial fission and fusion cycles are closely interconnected with cell metabolism and stress resistance^53^. In our work we tested the possible connections between age-dependent mitochondrial network reshaping and the accumulation of major pleiotropic drug resistance ABC-transporter Pdr5p in the plasma membrane. The possible regulatory connection between mitochondrial dynamics and *PDR5* expression has been recently found^54^. Earlier, it was found that mitochondrial dysfunction activates pleiotropic drug resistance^28,55^. Importantly, in the screening based on the method of transposon mutagenesis for *PDR5* activators, three out of five insertions were found in yeast mitofusin gene *FZO1* ^55^. The product of this gene is responsible for mitochondria fusion. However, we found that in the case of membrane proteins Pdr5p and Can1p the significant mother-to-bud asymmetry did not lead to a significant trends of the protein levels changes in the mother cells (Figs 4 and 5). We explain these results by the orchestrated proteostasis of these plasma membrane proteins which compensates the possible variability of *PDR5* expression that can be induced by disorganized mitochondrial network. For instance, it was shown that Pdr5p and Can1p can be degraded via ubiquitin-dependent endocytosis^56,57^. Otherwise, the age-dependent difference of mitochondrial network during early phases of yeast replicative aging could be an epiphenomenon, which does not affect any parameters determining cell fitness.

Newborn daughter cells emerge with the reset replicative lifespan, while deleterious processes are expected to randomly affect the cell systems with age. Therefore, we expected an increased variation of stress-response proteins levels in replicative age cohorts of elder mother cells compared to daughter cells or young mothers. We also hypothesized that bet hedging of *PDR5* expression level could be a reasonable strategy for cells to survive possible environmental stresses. However, we did not find any drift in Pdr5-GFP levels in yeast mother cells with age. Moreover, we found that the coefficient of variation of Pdr5-GFP levels was significantly lower in the mother cells with replicative age above five if compared to the daughter cells (Fig. 4c). This data indicates that individual variation of Pdr5 levels is achieved only at the level of mother-bud asymmetry, whereas in mother cells the levels are stable.

To summarize, we compared the level of five yeast proteins and one heterologous protein in different replicative age cohorts of yeast cells. Our results showed that cell-to-cell variations in the expression of these proteins are mostly due to the mother-bud difference. We, however, confirmed the previous reports showing that mitochondrial network morphology decreases its interconnectivity already during early replicative aging. Also a small age-dependent decrease in Idh1p and Idh2p levels was found for mother cells. This result suggests that a decrease in isocitrate dehydrogenase subunits levels may cause the early age-dependent decline in TCA cycle capacity. At the same time, the coefficients of variation of Idh1-GFP for mother and daughter cells were not significantly different (according to Fligner-Killeen test). Therefore, our results did not provide any strong evidence for the hypothesis of mother cell age-specific differentiation. Probably a study of less abundant proteins will show higher correlations with mother cell age. We suggest that changes related to mitochondria — organelles which have some autonomy from the nuclear regulation — can initiate the differentiation in asymmetrically divided eukaryotes.

## Material and Methods

### Yeast strains and growth conditions

In the study we used *TRX2-GFP*, *PDR5-GFP*, *IDH2-GFP* and *CAN1-GFP* yeast strains from yeast GFP clone collection^58^. *IDH1-GFP* (*IDH1-GFP LEU2 TRP1 W303-1A*) and *103Q-CFP* (P_GAL_-*103Q-CFP HIS3 W303-1A*) were constructed earlier in our laboratory in W303 mat *a* genetic background^40,59^. Yeast strains were grown in YPD (yeast peptone d-glucose) liquid medium with the exception of *103Q-CFP* strain. *103Q-CFP* was grown in YPRaf (yeast peptone raffinose), the expression of P_GAL_-*103Q-CFP* was induced by replacement of YPRaf with YPRafGal (yeast peptone raffinose and galactose) medium for five hours. Yeast media (YPD, YPRaf, YPRafGal) were prepared as described by Sherman^60^.

### Microscopy

For microscopy yeast cells were grown overnight on solid YPD medium, then resuspended in 200 µl PBS buffer, stained with with TRITC-ConA (tetramethylrhodamine conjugate of concanavalin A, 50 µg/ml) and incubated for 10 minutes at room temperature, then washed twice with PBS buffer, added to 5 ml liquid YPD medium and cultivated for 6 hours (unless indicated otherwise) to allow 4–5 rounds of budding. Importantly, TRITC-ConA does not affect GFP fluorescence (Fig. S4). One could argue that the observed decrease of Idh1-GFP with age is a result of the transition from solid to liquid medium. However, we obtained the similar results in the experiments with prolonged preincubation in liquid YPD medium (Figure S5). For stress induction, 2 mM H_2_O_2_ or 20 µM clotrimazole were added and the cells were incubated for additional 2–3 hours. Thereafter cells were stained with 5 µM calcofluor white (CW) in order to count the bud scars. Then the cells were washed to remove the medium and resuspended in PBS buffer. Fluorescence microscopy was carried out using Olympus BX51 upright microscope, UPLANFLN 100x lens (N.A. 1.3) with the set of filters presented in Table S1. Images were taken with Olympus DP30BW CCD camera using Olympus CellF software. Color photographs of the cells were generated by pseudocoloring: GFP-channel black and white image was copied into green channel of RGB file.

We imaged cells in GFP-channel and afterwards were switching to CW-channel to count the bud scars. Imaged cells were ranged by their replicative age as follows: a single cell without any bud scars or a bud larger than half of the volume of it’s mother cell were counted as ‘daughter cells’, a single cell with one bud scar or a cell without a bud scar but with a bud - as age 1, etc. During each experiment we imaged 50 cells at random (i.e. it can be considered as random sampling from yeast population) and 50 TRITC-positive cells to enrich data with the old mother cells.

GFP levels in cells were measured using ImageJ software. Average signal intensity was measured for each cell, background signal intensity was subtracted. For each experiment the individual cell signal intensity was normalized to the population mean. The latter step was necessary due to the moderate differences between separate day experiments that were probably due to the instability in the mercury lamp excitation intensity.

In case of Trx2-GFP, Idh1-GFP, Idh2-GFP and 103Q-CFP we analyzed the whole cell fluorescence signals. To determine Pdr5-GFP and Can1-GFP levels, we measured the signal only within the plasma membrane area (0.25 µm [4 px] wide cell perimeter, Fig. S1). We used GIMP and Inkscape to crop and arrange the figure panels.

### Mitochondrial morphology assay

Cells expressing Idh1-GFP were photographed in GFP-channel (see Table S1) and the number of bud scars was counted and recorded in CW-channel. Next, photograph of each individual cell was attributed to one of three mitochondrial morphology classes: fused, intermediate or fragmented. Attribution was made by visual similarity between sample photographs of each aging class (Fig. 2a inset, Figure S3). Importantly, attribution was made by experimentator who did not know the replicative age of each cell. As a result, the table of individual cells with age and mitochondrial morphology attribute was generated.

### Flow cytometry

For FACS experiments yeast cells were grown overnight in liquid YPD medium to 4 × 10^7^ cells/ml, then the stress was induced by 2 mM H_2_O_2_ or 20 µM clotrimazole followed by 2–3 hour incubation. Yeast cells were washed and resuspended in PBS buffer. To quantify GFP fluorescence we performed flow cytometric analysis using CytoFLEX flow cytometer (Beckman Coulter). All flow cytometry data were analyzed with CytExpert software (Beckman Coulter). At least 10000 cells were analyzed per experiment.

### Data Analysis and visualization

Each data set (i.e. strain, conditions) was analyzed first by Kruskall-Wallis test. Each age cohort of yeast cells was considered as separate experimental condition for this test. None of the tested conditions showed any significance if only mother cells were taken for analysis. If a result of Kruskall-Wallis test (including daughter cells) was significant (P < 0.05), than Nemenyi test was applied and all P values below cut-off threshold (P < 0.05) were indicated on the figures. For Nemenyi test mother cells with replicative age above 8 were considered as a single condition. Chi-squared contingency table test with Monte Carlo simulation (50000 replicates) was used to analyze difference in mitochondrial morphology between different age cohorts. Fligner-Killeen Test of Homogeneity of Variances was used to test of the null hypothesis that the variances in each of the age cohorts are the same. Cells that were found using TRITC-ConA visual enrichment were analyzed separately from the cells that were photographed without visual selection procedures. Data is plotted as boxplots with boxes widths proportional to the square-roots of the number of observations in the groups. Tests were conducted using R. Data were visualized with R or QtiPlot.

### Data Availability

The datasets generated and/or analysed during the current study are available from the corresponding author on reasonable request.

## Electronic supplementary material

Supplementary Information
