# Supplementary material for: The contribution of Saccharomyces cerevisiae replicative age to the variations in the levels of Trx2p, Pdr5p, Can1p and Idh isoforms
Source: Sci Rep. 2017 Oct 16;7:13220. doi: 10.1038/s41598-017-13576-w (PMC5643315; doi:10.1038/s41598-017-13576-w)
Supplement: Supplementary file 1 — Supplementary Information [file 41598_2017_13576_MOESM1_ESM.pdf]

SUPPLEMENTARY MATERIALS

The contribution of *Saccharomyces cerevisiae* replicative age to the variations in the levels of Trx2p, Pdr5p, Can1p and Idh isoforms

Aglaia V. Azbarova[1,2], Kseniia V. Galkina[1], Maxim I. Sorokin[2,3,4], Fedor F. Severin[2], Dmitry A. Knorre[2,*]

[1]*Faculty of Bioengineering and Bioinformatics, Moscow State University, Leninskiye Gory 1-73, Moscow 119991, Russia*

[2]*Belozersky Institute of Physico-Chemical Biology, Moscow State University, Leninskiye Gory 1-40, Moscow 119991, Russia*

[3]*National Research Centre Kurchatov Institute, Centre for Convergence of Nano-, Bio-Information and Cognitive Sciences and Technologies, Moscow 123182, Russia*

[4]*OmicsWay Corp., 340 S Lemon Ave, Walnut, CA, 91789*

**** knorre@belozersky.msu.ru***

Table S1. Set of fluorescent filters used.

In text	*Name*	*Exciter filter, nm*	*Beam splitter, nm*	*Barrier filter, nm*
CW-channel	U-MNU2	360-370	400	420
GFP-channel	U-MNG2	530-550	570	590
CFP-channel	U-N31044v2	426-446	455	460-500
TRITC-channel	U-MNIBA3	470-495	505	510-550

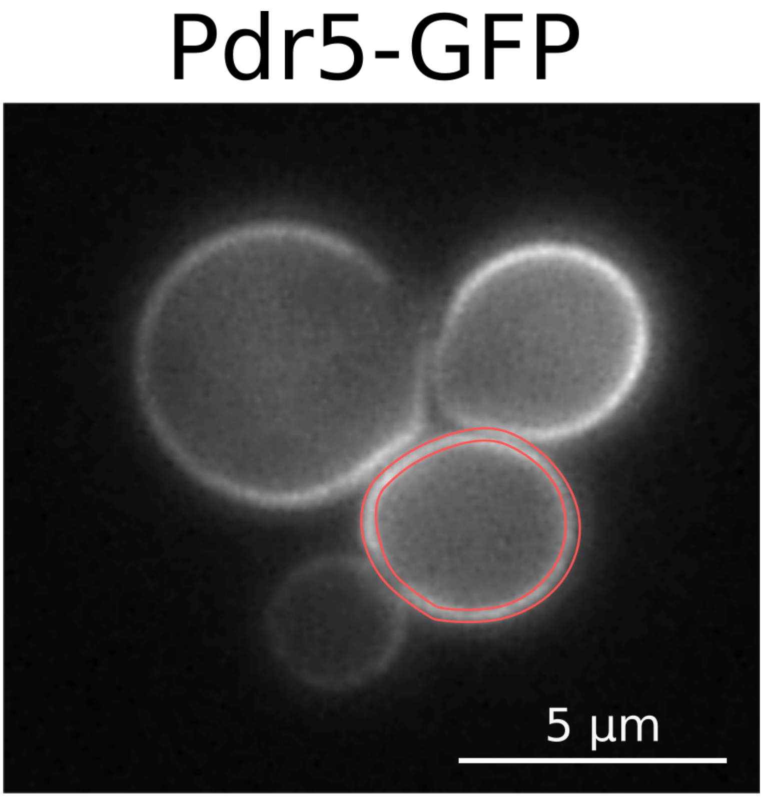

Figure S1. Yeast cells expressing Pdr5-GFP. Red outline indicates a representative area of the cell that was analyzed in the experiments with Pdr5-GFP and Can1-GFP.

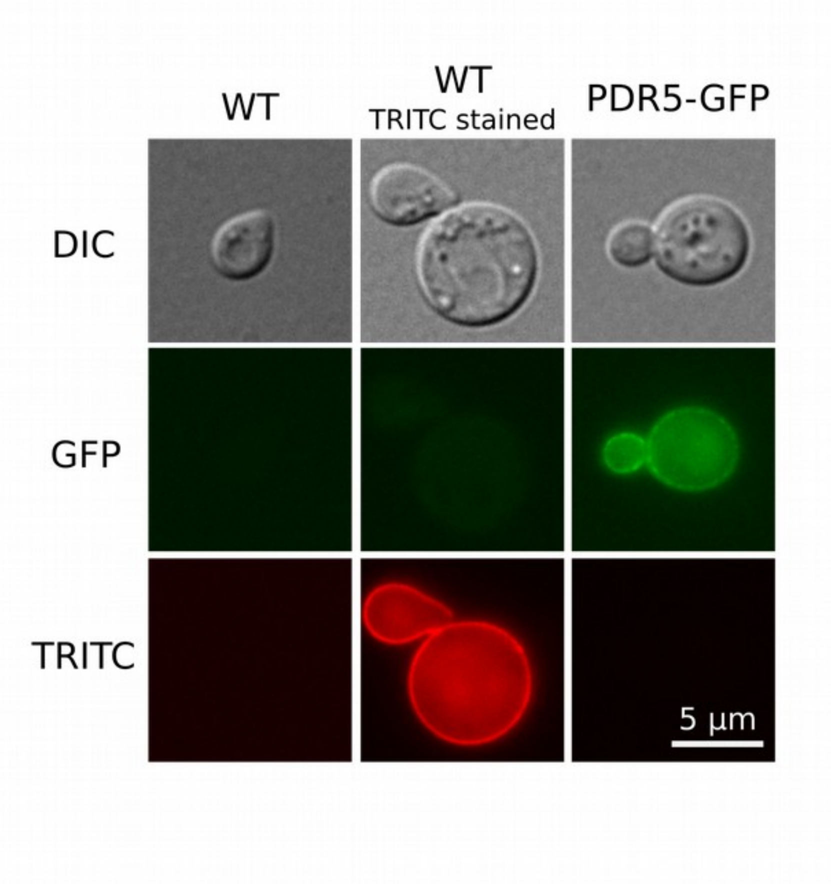

Figure S2. The absence of TRITC-ConA fluorescence leakage in U-MNIBA3 filter set used for GFP detection.

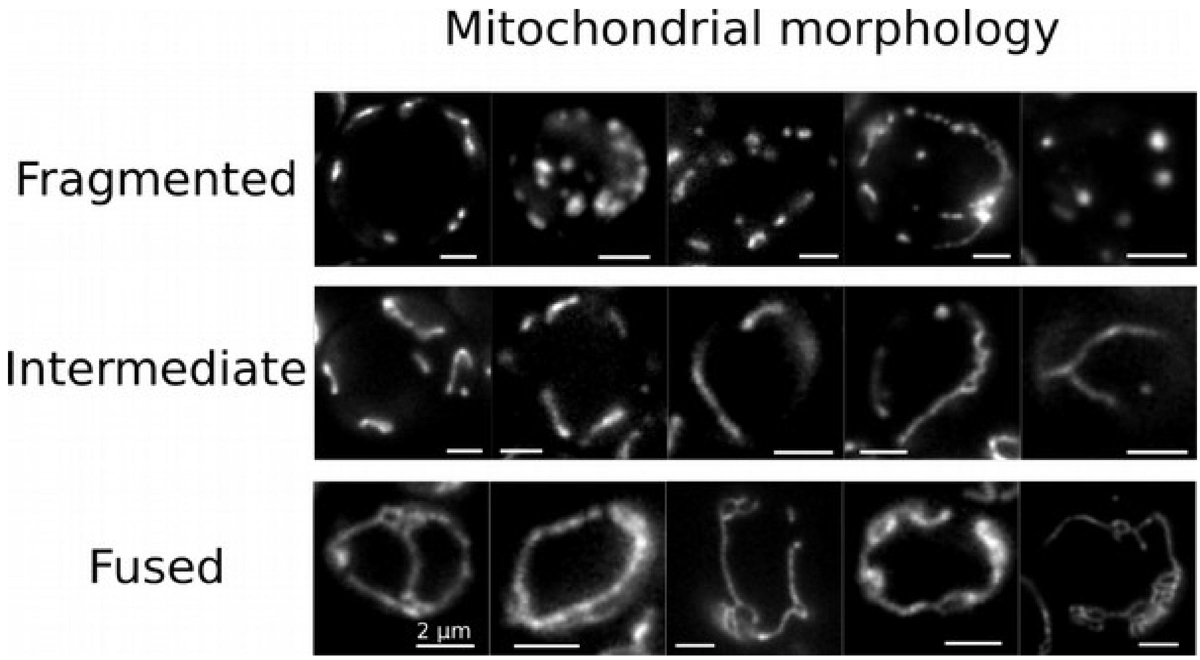

Figure S3. Yeast cells expressing Idh1-GFP. Representative photographs of mitochondrial morphology classes used as reference for mitochondrial morphology annotation. All scale bars are 2 µm.

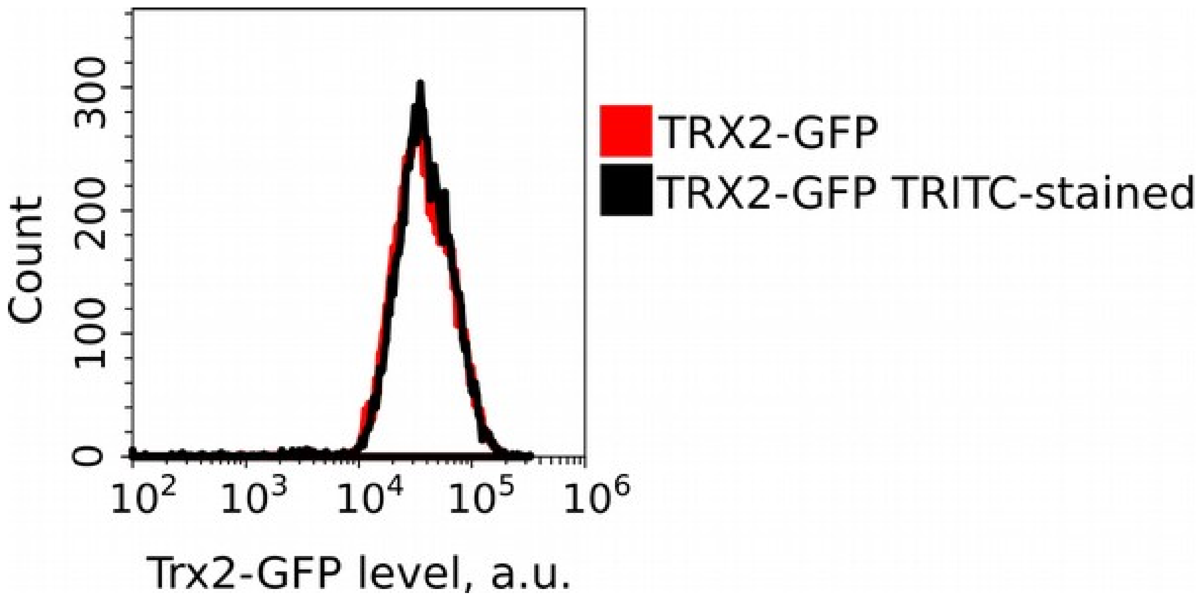

Figure S4. Staining with TRITC-ConA does no effect on Trx2-GFP fluorescence. Analysis of Trx2-GFP levels by flow cytometry under the control conditions and after TRITC-ConA staining. A.u. — arbitrary units.

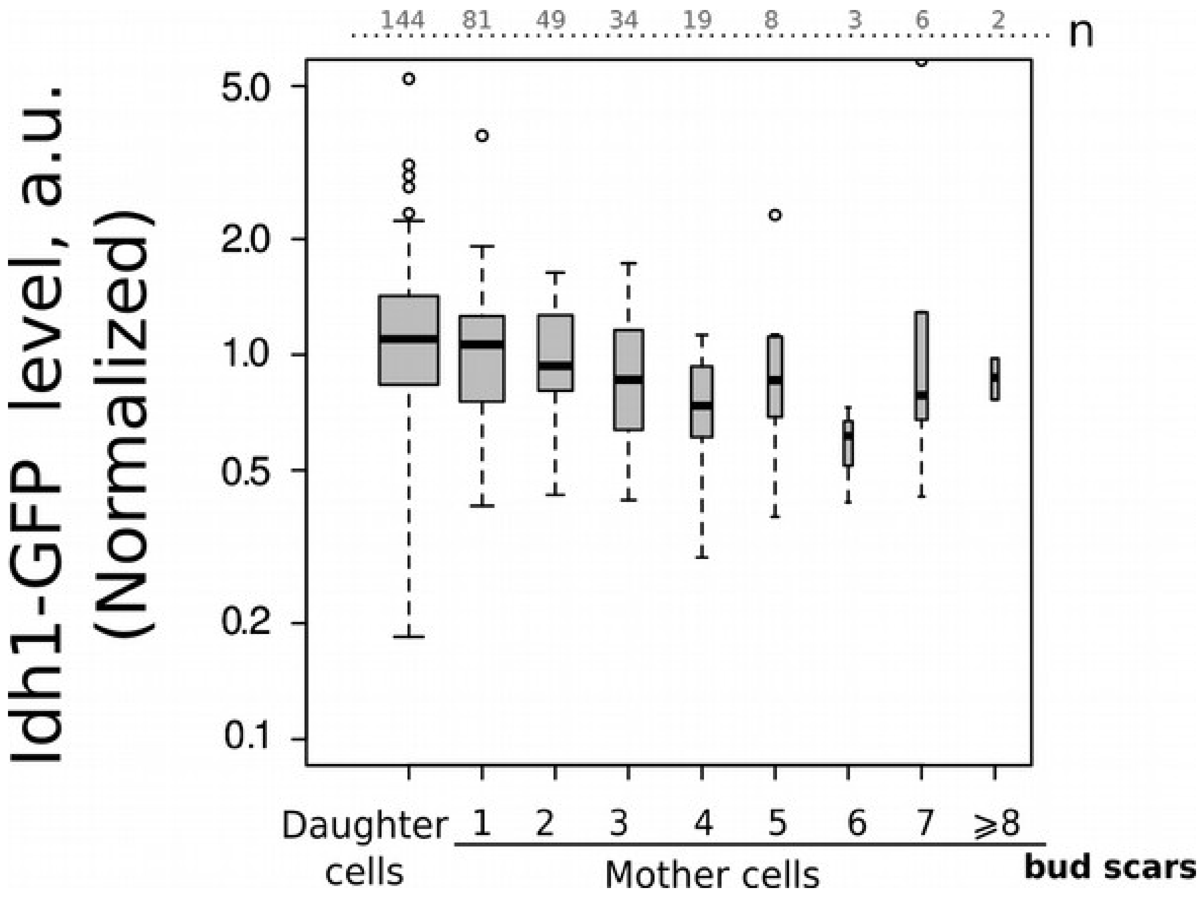

Figure S5. Idh1-GFP levels in individual cells of different replicative age cohorts incubated in liquid YPD for 10 hours. The numbers of analyzed cells for each age class are shown at the top of the boxplot. Kendall's *tau* for correlation of protein level with replicative age of mother cells was equal to -0.17 (p = 0.001138). a.u. — arbitrary units.
